# Polymeric Inclusion Membranes Based on Ionic Liquids for Selective Separation of Metal Ions

**DOI:** 10.3390/membranes13090795

**Published:** 2023-09-13

**Authors:** Adrián Hernández-Fernández, Eduardo Iniesta-López, Anahí Ginestá-Anzola, Yolanda Garrido, Antonia Pérez de los Ríos, Joaquín Quesada-Medina, Francisco José Hernández-Fernández

**Affiliations:** 1 Department of Chemical Engineering, Faculty of Chemistry, University of Murcia (UMU), P.O. Box 4021, Campus de Espinardo, E-30100 Murcia, Spain; adrian.h.f@um.es (A.H.-F.); eduardo.iniestal@um.es (E.I.-L.); ygh46006@um.es (Y.G.); aprios@um.es (A.P.d.l.R.); quesamed@um.es (J.Q.-M.); 2 Technological Center for Energy and Environment, E-30353 Cartagena, Spain; anahi.ginesta@cetenma.es

**Keywords:** separation, metal ions, ionic liquids, polymer inclusion membranes, ionogel, stability

## Abstract

In this work, poly(vinyl chloride)-based polymeric ionic liquid inclusion membranes were used in the selective separation of Fe(III), Zn(II), Cd(II), and Cu(II) from hydrochloride aqueous solutions. The ionic liquids under study were 1-octyl-3-methylimidazolium hexafluorophosphate, [omim^+^][PF_6_^−^] and methyl trioctyl ammonium chloride, [MTOA^+^][Cl^−^]. For this purpose, stability studies of different IL/base polymer compositions against aqueous phases were carried out. Among all polymer inclusion membranes studied, [omim^+^][PF_6_^−^]/PVC membranes at a ratio of 30/70 and [MTOA^+^][Cl^−^]/PVC membranes at a ratio of 70/30 were able to retain up to 82% and 48% of the weight of the initial ionic liquid, respectively, after being exposed to a solution of metal ions in 1 M HCl for 2048 h (85 days). It was found that polymer inclusion membranes based on the ionic liquid methyl trioctyl ammonium chloride allowed the selective separation of Zn(II)/Cu(II) and Zn(II)/Fe(III) mixtures with separation factors of 1996, 606 and, to a lesser extent but also satisfactorily, Cd(II)/Cu(II) mixtures, with a separation factor of 112. Therefore, selecting the appropriate ionic liquid/base polymer mixture makes it possible to create polymeric inclusion membranes capable of selectively separating target metal ions.

## 1. Introduction

Some human activities release harmful substances into water sources, posing significant risks to aquatic ecosystems and the environment [1]. Among the contributors to water pollution, heavy metal ions stand out due to their high toxicity, non-degradability, and potential to bioaccumulate and biomagnify in the food chain [2]. The presence of heavy metal ions in aquatic ecosystems can have direct or indirect detrimental effects on living organisms [3]. Moreover, these ions also threaten plants and animals in soil environments, as they can be absorbed by plants and subsequently reach animals and humans [4].

Industrial and academic researchers are dedicating their efforts to encouraging the recycling of waste materials for their utilization in various industrial applications [5]. This practice of recycling waste materials contributes to mitigating the environmental impact of industrial activities and helps conserve natural resources. Certain wastes exhibit a metallic nature, as exemplified by the spent pickling hydrochloric acid effluent generated by the galvanic industry. This effluent contains high levels of Zn(II), Fe(III), and small quantities of other heavy metals [6]. By recycling this effluent, the metals can be recovered and subsequently reused in other industrial processes [7].

Electronic devices represent yet another significant source of metallic waste [8]. Burning these devices comes with significant dangers for both the environment and human well-being [9]. Within the European Union, incinerating electronic waste has been calculated to release 36 tons of mercury and 16 tons of Cadmium into the atmosphere annually [10]. The solid waste stemming from metallurgical zinc industries predominantly comprises copper. This procedure generates a sludge rich in copper, accompanied by other metal ions like iron and cadmium. The extraction and isolation of these metal ions from their mixed solutions hold substantial importance in hydrometallurgical processes due to the potential for reclaiming valuable metals and addressing environmental pollution issues [11].

Numerous technologies exist for eliminating toxic metals from liquid effluents, such as adsorption, membrane, chemical, electric, and photocatalytic-based treatments [12]. However, many of these methods are complex and require significant energy input and/or production of large quantities of waste [13].

Membrane-based separation processes have emerged as a hopeful substitute for addressing the limitations of conventional separation techniques, including high energy usage and the necessity for severe operating conditions. These processes operate without energy consumption and can be carried out under more moderate conditions [14,15].

Supported liquid membranes (SLMs) have gained increasing interest as a membrane-based separation technique over the past few decades. SLMs involve incorporating an organic liquid into the tiny pores of polymeric support, where capillary forces retain it [16]. The benefits of SLMs encompass the utilization of a small volume of organic solvent and carrier compared to liquid-liquid extraction, enabling the use of costly carriers, simultaneous extraction and re-extraction steps, achieving a high separation factor, ease of scaling up, low energy demands, operation under modest conditions, and cost-effectiveness in terms of both capital and operating expenses [16]. Enhancing the stability of SLMs can be achieved through a novel approach that employs ionic liquids as the liquid phase [17]. This innovative approach has found practical applications in selectively transporting various organic compounds [18], metal ions [19] and inorganic salts [20]. Ionic liquids (ILs) are organic salts characterized by their ability to remain liquid below room temperature [21]. Typically, ILs are composed of an organic cation paired with a monoatomic or polyatomic inorganic anion, though the trend is shifting towards using organic anions. One of the primary advantages of ILs compared to organic solvents is their remarkably low vapor pressure, rendering them highly stable.

Furthermore, ionic liquids (ILs) showcase advantageous chemical and thermal stability and high viscosity [22]. Their solubility within neighboring phases can be meticulously regulated by selecting the appropriate cation and anion [13]. The distinctive attributes of ionic liquids allow them to be used as an extracting phase in ion metal liquid-liquid extraction [23] or even novel liquid-liquid extraction approaches [24]. Ionic liquid properties also contribute to the establishment of a more enduring membrane phase. Supported liquid membranes (SLMs) that incorporate ionic liquids are named supported ionic liquid membranes (SILMs) [17].

Over the last years, various categories of ionic liquid membranes have emerged. Among them, polymer ionic liquid inclusion membranes (PILIMs) have garnered considerable attention for their potential in diverse chemical operations and applications like metal ion extraction, salts separation, CO_2_ removal or selective electrodes [25,26,27,28]. However, much fewer examples of PILIMs regarding SILMs could be found in literature due to their most recent appearance. PILIMs are characterized by entrapping the ionic liquid within a polymeric structure, effectively reducing its release into the surrounding phase. PILIM_S_ is also known as ionogels. While both SILMs and PILIMs operate on similar separation mechanisms, PILIMs stand out due to their superior mechanical and chemical properties [29]. This advantage is due to the immobilization of the ionic liquid within a polymeric matrix, setting them apart from supported membranes [29]. Previous research has shown the high stability of PILIMs based on [omim^+^][PF_6_^−^] [29]. Likewise, some studies place [omim^+^][PF_6_^−^] and [MTOA^+^][Cl^−^] ionic liquids as good liquid-liquid phase extracting agents for metals such as Cu(II), Fe(III), Zn(II) and Cd(II) [26] and in the form of ionic liquid membranes for the same purpose [19,30]. These findings suggest that employing ionic liquids as a substitute for conventional extraction agents in the selective separation of heavy metal ions holds significant promise.

The present works aim to explore the feasibility and efficiency of using PILIMs for the extraction and separation of Zn(II), Cd(II), Fe(III), and Cu(II) from hydrochloride aqueous solution in the absence of a chelating agent. [omim^+^][PF_6_^−^] and [MTOA^+^][Cl^−^] were used as ionic liquid phase. Their stability against hydrochloric and aqueous solutions has been tested, as well as their metal ion separation efficiency.

## 2. Methods

### 2.1. Chemicals

Zn(II), Cd(II), Cu(II) and Fe(III) hydrochloride aqueous solutions were prepared by dissolving ZnCl_2_, CdCl_2_, CuCl_2_ and FeCl_3_·6H_2_0, all analytical grade (from Sigma Aldrich Fluka Chemical Co., Madrid, Spain), in hydrochloric acid (1 M from Panreac Química S.A., Madrid, Spain) at 100 mg/L. The polymers poly(vinylchloride) (PVC) and the ionic liquid methyl trioctyl ammonium chloride, [MTOA^+^][Cl^−^] (purity > 97%) were purchased from Sigma Aldrich Fluka Chemical Co., Madrid, Spain. The ionic liquid 1-octyl-3-methylimidazolium hexafluorophosphate ([omim^+^][PF_6_^−^]) (purity > 99%) was purchased from Iolitec GmbH (Denzlingen, Germany). Other solvents and chemicals used in the research were acquired from Panreac Química S.A. with the highest purity available (Madrid, Spain).

### 2.2. Polymer Ionic Liquid Inclusion Membranes Preparation

Different mixtures of an ionic liquid and a polymer were dissolved in 3 mL of different solvents to prepare 0.3 g polymer ionic liquid inclusion membranes of different compositions. The casting method, described in other articles [29], was used to create the different PILIMs by pouring the prepared mixtures into a Fluka glass ring (30 mm height, 28 mm internal diameter) placed on a borosilicate glass plate and allowing all the solvent to evaporate for 48 h.

### 2.3. Analysis of Membrane Stability

The new polymer inclusion membranes’ stability toward an aqueous phase was assessed by immersing the new PILIMs in a continuously agitated (300 rpm) 60 mL water bath set at 30 °C. Each experiment comprised four 24-h cycles, followed by a 24-h drying period and subsequent weighing of the membranes. Before commencing the next cycle, the aqueous phase was changed with fresh water. To analyze the stability of the PILIMs, the weight loss of the ionic liquid was measured by weighting the membrane before and after each cycle, which comprises 24 h.

### 2.4. Membrane Transport Studies

**Figure 1:**
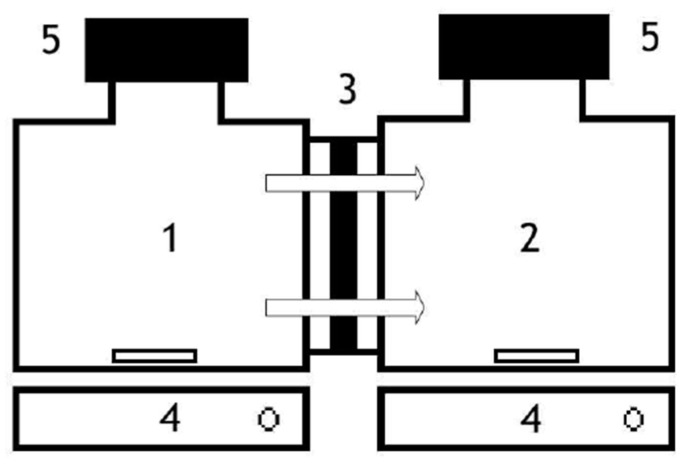
Diffusion cell discontinuous set-up used for experiments: (1) feed solution; (2) receiving solution; (3) PILIM; (4) magnetic stirrer; and (5) septum.

We assessed the transportation of Zn(II), Cu(II), Cd(II), and Fe(III) through the PILIMs at a temperature of 303.15 K using the setup depicted in Figure 1. The setup consisted of a glass diffusion cell with two separate compartments containing 30 mL of solution and separated by the PILIM. Both compartments were subjected to mechanical stirring at 300 rpm to avoid concentration polarization conditions at the membrane interfaces, which improves the extraction conditions and reduces the instability of the membranes, as recommended in previous works [31]. The receiving phase was composed of MilliQ water. Once 30 mL of the respective solutions had been added to each compartment, the experiment was started. Atomic absorption spectrophotometry was chosen to monitor the assay, performing periodic analyses by taking 100 µL from each compartment, as described in Section 2.5. Sampling was concluded once the concentrations of the metal ions in both phases had stabilized.

The pertraction factor (PF) was the variable used to evaluate the efficacy of the pertraction procedure. Its value was determined by applying the equation (Equation (1)) as follows:

(1) $$PF=\frac{C_{rM}}{C_{fM}}$$

where $C_{rM}$ represents the concentration of the metal ion in the receiving phase and $\;C_{fM}$ in the feed phase. To ensure the repeatability of the assay, triplicate determinations were performed, and the resulting mean values were presented. The assay showed a high level of repeatability, with a relative standard deviation of 3% or less.

To evaluate the ability of the PILIMs to separate target metal ions, a separation factor ($\alpha_{M1/M2}$) was determined as follows (Equation (2)). This factor allows us to know the efficiency of the process to separate the metal ions by specific PILIMs. The higher $\alpha_{M1/M2}$, the more selective the separation by the specific PILIMs.

(2) αM1/M2=CrM1CfM1CrM2CfM2

where $C_{{rM}_{1}}$ and $C_{{fM}_{1}}$ represent the concentration of the metal ion M1 in the receiving and feed phase, respectively, and $C_{{rM}_{2}}$ and $C_{{fM}_{2}}$ represent the concentration of the metal ion M2 in the receiving and feed phase, respectively.

### 2.5. Analytical Method

The determination of metal ions concentrations, including Zn(II), Cd(II), Cu(II), and Fe(III), was carried out using a Varian, Spectra 10 Plus model, atomic absorption spectrophotometer. As an internal emission source, a specific hollow cathode lamp for zinc, cadmium, iron and copper is used. The calibrating of the equipment was carried out using metal ion standards at concentrations of 0, 0.1, 0.5, 1, 1.5 and 2 mg/L. from a commercial standard of 1000 mg/L. The correlation coefficient of the calibration curves (r^2^) was greater than 0.99. The working conditions were oxidant: air (3.5 mL/min) and fuel: acetylene (1.5 mL/min). To monitor the sorption of metal ions, periodic samples were withdrawn from the aqueous solutions for analysis.

## 3. Results

### 3.1. Stability of Polymer Ionic Liquid Inclusion Membranes Based on [omim^+^][PF_6_^−^] and [MTOA^+^][Cl^−^] to Aqueous Medium/Hydrochloride Solutions

Water’s widespread use as a universal solvent is attributed to its polarity. Research studies have shown that some supported ionic liquid membranes, for instance, those based on nylon support, exhibit instability when they come into contact with polar solvents like water [17]. The immobilization of the ionic liquid as a polymeric inclusion ionic liquids membrane could enhance the stability of the ionic liquids membrane and could increase the amount of active phase (ionic liquids) in the membrane.

In this work, the stability of PILIMs based on [omim^+^][PF_6_^−^] and [MTOA^+^][Cl^−^] at different ionic liquid/PVC ratios toward aqueous medium have been investigated. [omim^+^][PF_6_^−^] and [MTOA^+^][Cl^−^] were selected because of their low water solubility [29,32]. The low solubility of ionic liquids in the surrounding phase has been demonstrated to be a crucial factor in the stability of PILIMs [17].

**Figure 2:**
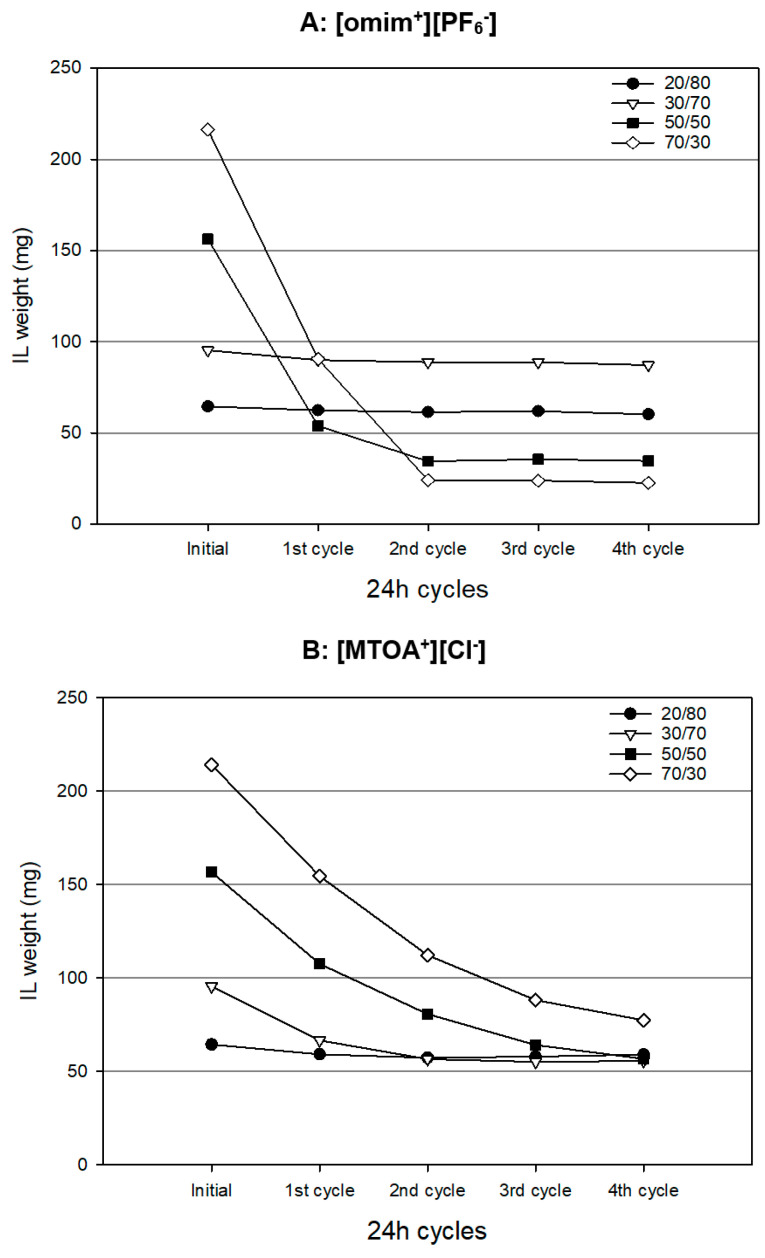
Profile of the weight losses of ionic liquid in PILIMs after each cycle in contact with deionized water for different ionic liquid/PVC ratios. (**A**) [omim^+^][PF_6_^−^] (**B**) [MTOA^+^][Cl^−^].

Figure 2 shows the profile of the weight losses of ionic liquids from the membranes prepared with different IL/PVC ration during four cycles in contact with fresh water in each cycle. It can be observed that the percentage of retained ionic liquid increases as the proportion of the base polymer increases. The variations of weight observed after four cycles are attributed to the losses of IL (ionic liquid) due to PVC’s insolubility in water. The results indicate a rise in IL losses when using high [omim^+^][PF_6_^−^] to PVC ratios. This fact implies that each amount of PVC can only retain a specific quantity of IL. Furthermore, an increase in the initial amount of IL used in the PILIM does not necessarily result in a higher amount of IL retained.

Figure 2A illustrates the behavior of the membrane based on [omim^+^][PF_6_^−^], where the retained amount of ionic liquids is stabilized after the second cycle for higher concentrations of ionic liquids (50% and 70%). The membrane prepared with 30% IL achieves the highest retained amount of this ionic liquid at the end of the test.

In the case of the PILM based on [MTOA^+^][Cl^−^] (Figure 2B), the retained amount of ionic liquid tends to stabilize after the fourth cycle, with approximately 55 mg for membranes prepared at 20%, 30% and 50%, and 77 mg for the membranes prepared at 70%.

The selection of membranes with the maximum retained amount of the active phase (ionic liquid) is essential for the pertraction process. The ionic liquid/PVC ratio determining the highest retention varies depending on the specific ionic liquid used. Therefore, the stability of the obtained membranes is influenced not only by the solubility of the ionic liquids but also by the specific interactions between the ionic liquids and the polymer, PVC, in this case. In water, [omim^+^][PF_6_^−^] exhibits greater solubility compared to [MTOA^+^][Cl^−^] [17,32], resulting in the need for a larger amount of PVC to immobilize a higher quantity of IL. In spite of the higher stability of PILIM membranes with 20%, 30%, and 50% with respect to 70% of [MTOA^+^][Cl^−^] (due to their higher ratio PVC/IL), 70% IL membrane was chosen for pertraction experiments due since this membrane contains the higher amount of immobilized active phase ([MTOA^+^][Cl^−^]). Furthermore, the use of less amount of PVC in [MTOA^+^][Cl^−^]/PVC at 70/30 (*w*/*w*%) could facilitate the transport of metal ions through the membrane, as explained above.

Based on the obtained results, membranes containing 30% [omim^+^][PF_6_^−^] and 70% [MTOA^+^][Cl^−^] were chosen for the pertraction test presented in the subsequent sections due to the higher amount of ionic liquids immobilized after four-cycle.

Considering that the feed phase in the pertraction tests consists of a hydrochloric acid solution, it is essential to assess the stability of these membranes against the hydrochloric acid aqueous medium. The stability tests were conducted using 60 mL of 1 M HCl at similar conditions to the pertraction assays.

**Figure 3:**
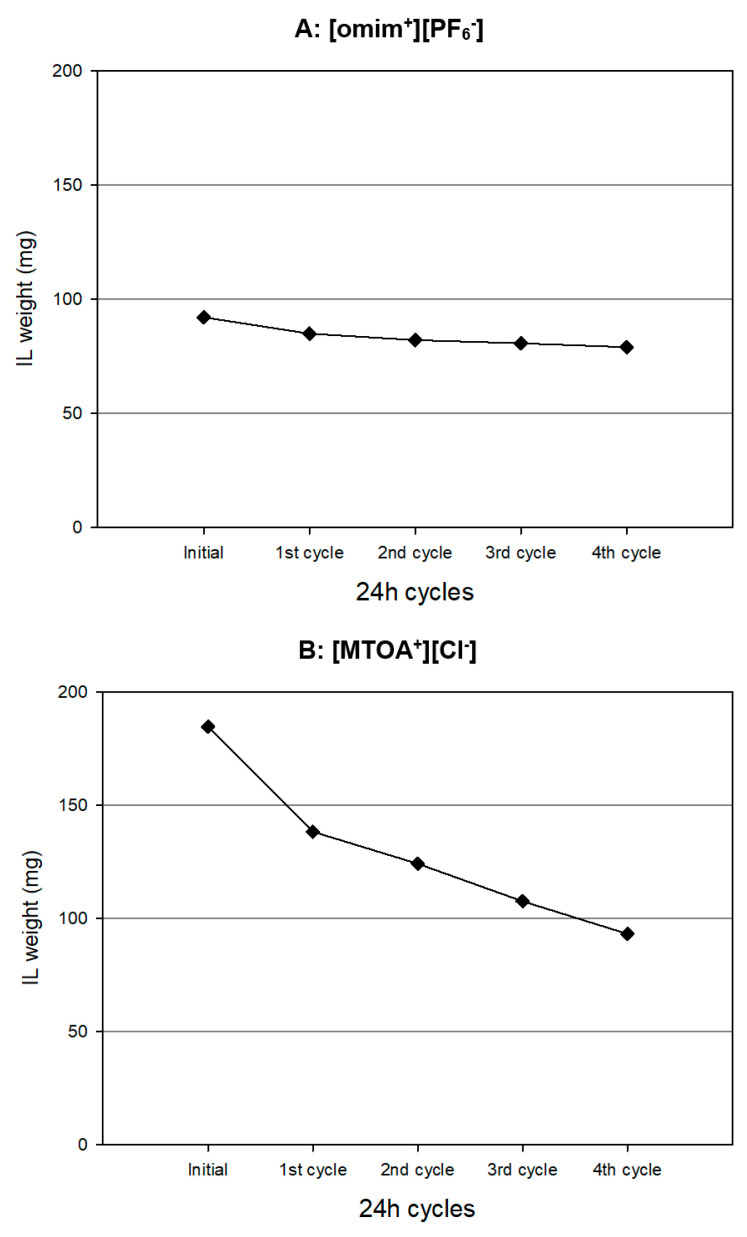
Profile of the weight losses of ionic liquid from PILIMs after each cycle in contact with the hydrochloric solution for different ionic liquids. (**A**) [omim^+^][PF_6_^−^]/PVC (30/70) (**B**) [MTOA^+^][Cl^−^]/PVC (70/30).

Figure 3 shows the profile of the weight losses of the ionic liquid of the membranes prepared with different IL/PVC ratios for [MTOA^+^][Cl^−^] and [omim^+^][PF_6_^−^] during the four cycles in contact with hydrochloric solution (1 M). In the case of the ionic liquid [omim^+^][PF_6_^−^], the PIM exhibits good stability. Although more ionic liquid is lost during the cycles compared to when the membrane is in contact with pure water, approximately 80% of the initial ionic liquid is retained. Conversely, with the IL [MTOA^+^][Cl^−^], a significant loss of the IL is observed during each cycle. However, the loss of ionic liquid is less than in pure aqueous solution as a phase.

Furthermore, in this case, the amount of the final active phase in the membrane is higher than that in the membrane based on [omim^+^][PF_6_^−^] at 30%. Notably, the stability of [MTOA^+^][Cl^−^] based PIMs improves when exposed to hydrochloric acid solutions. The existence of the same counteranion [Cl^−^] in the aqueous phase (HCl, 1 M) could stabilize the ionic liquids in the polymer inclusion membrane.

The use of casting techniques to immobilize ionic liquids in PIMs enables the production of resilient PILIMs capable of enduring hydrochlorinated aqueous environments, even with a loading capacity of up to 79 mg (232 mM) and 93.2 mg (231 mM) for [omim^+^][PF_6_^−^] and [MTOA^+^][Cl^−^], respectively, after four-cycle operation which involves a loading capacity of up to 12.8 and 15.1 mg IL/cm^2^ (37.6 and 37.4 mM IL/cm^2^) for [omim^+^][PF_6_^−^] and [MTOA^+^][Cl^−^], respectively. Practically, the same millimoles of [omim^+^][PF_6_^−^] were immobilized as [MTOA^+^][Cl^−^], with the difference that in the case of B, less PVC (30%) was needed for immobilization. It is important to highlight that optimal ionic liquid concentration will depend on the ionic liquid nature and specifically on the ionic liquid water solubility and interaction with the organic polymer support, in our case, PVC. In previous work, polymeric Nylon membranes of 25 mm diameter were used to immobilize by adsorption of different ionic liquids based on imidazolium cation to create supporting ionic liquid membranes (SILMs). The maximum ionic liquid immobilizes in the membrane pores before the stability experiment was around 90 mg of ionic liquids, which involves a loading capacity of up to 18.3 mg IL/cm^2^. After seven days of stability experiments, almost all the ionic liquid immobilized in the membrane was lost (from 98.5% to 100%) when high polar solvents (DMSO and water) were used in the receiving phase [17].

### 3.2. Selective Separation of Fe(III), Zn(II), Cd(II) and Cu(II) HCl 1 M Aqueous Solution trough PILIMs Based on [omim^+^][PF_6_^−^] and [MTOA^+^][Cl^−^] Using Mili-Q Water as Receiving Phase

As mentioned above, [omim^+^][PF_6_^−^] and [MTOA^+^][Cl^−^] have shown good results in terms of metal ion separation in other studies in liquid-liquid extraction and the form of ionic liquid-supported membranes [19,23]. However, there are currently no studies that have reported the use of polymeric inclusion membranes based on these ionic liquids to separate metal ions in hydrochloride solutions. In this study, we have used polymer inclusion membranes (ionogel) based on [omim^+^][PF_6_^−^] and [MTOA^+^][Cl^−^] at 30% and 70%, respectively ionic liquid concentrations. These ionic liquid concentrations have been shown, as can be seen in the previous section, as the best concentration to obtain a more stable and higher ionic liquid charge in polymer ionic liquid membranes.

#### 3.2.1. Selective Separation through Polymer Inclusion Membrane Based on 1-Octyl-3-methylimidazolium Hexafluorophosphate, [omim^+^][PF_6_^−^]

**Figure 4:**
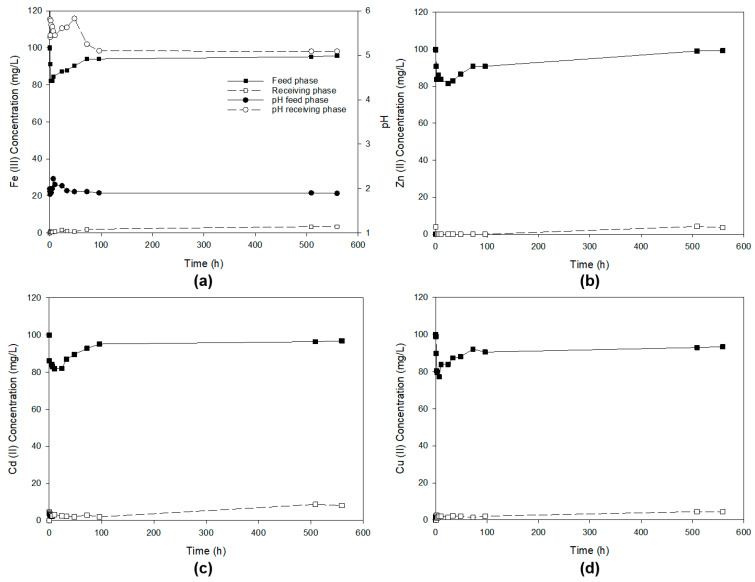
Metal ions concentrations and pH profiles (**a**) in the feed and receiving phases in the pertraction of Fe(III) (**a**), Zn(II) (**b**), Cd(II) (**c**) and Cu(II) (**d**) through a PILIM based on [omim^+^][PF_6_^−^]/PVC with 30% of IL. The feed phase was a mixture of the four metal ions at 100 mg/L in HCl (1 M). The receiving phase was milliQ water at pH = 6.

In this experiment, a mixture of the four metal ions at a concentration of 100 ppm, dissolved in 1 M HCl, constitutes the feed phase, while the receiving phase consists of milli-Q water with a pH equal to 6. Figure 4 shows the pH profile in the feed and acceptor phases and, separately, the concentrations of the metal ions in the pertraction studies of Fe(III), Zn(II), Cd(II) and Cu(II) from a 1 M HCl solution. Different concentrations of HCl were used as the driving force for ion pertraction.

As can be seen in Figure 4, during the initial moments of the experiments (0–100 h), there is a small decrease in the metal concentrations in the feed phase, but this does not translate into a quantitative increase in the concentration of the metal ions in the receiving phase. Instead, the initial concentration in the feed phase is practically reestablished. The same thing happens with the pH profiles. They decrease and increase in consonance with the concentration of the ions in their respective phases. Hence, the driving forces for transport could arise from differences in both metal ion concentration and HCl concentration between the feeding and receiving phases. In previous work [23], we studied the selective recovery of Zn(II), Cd(II), Cu(II) and Fe(III) from hydrochloride aqueous solutions (0.1 g/L, HCl-1 M). The extraction capability of [omim^+^][PF_6_^−^] for these metals was the following: Cd (≈70%) > Zn (≈30%) > Fe (≈20%) > Cu(≈5%). The operation with a polymer inclusion membrane does not improve the results found in liquid-liquid extraction experiments. We should consider that in liquid-liquid extraction experiments, the ration of 1 M hydrochloride aqueous solutions to the IL phase was 1 to 1 (*v*/*v*). In the polymer inclusion membrane experiment, the ration of the feed phase to the ionic liquid phase was approximately 30,000/50 (*v*/*v*) (see comments stability before). The unfeasibility of these dialkyl imidazolium cation-based ionic liquid inclusion polymeric membranes is most likely due to the fact that the amount of extractant phase was insufficient to extract the metals present in the feed phase and that the higher amount of PVC needed, which hinders transport through the membrane.

In terms of the membrane’s stability following the pertraction test, the observed outcome was not as anticipated. Surprisingly, the membrane retained only 48% of the initial ionic liquid, significantly lower than expected based on the earlier stability tests. This suggests that a high metal concentration could have an adverse effect on the stability.

#### 3.2.2. Selective Separation through Polymer Inclusion Membrane Based on Methyl Trioctylammonium Chloride, [MTOA^+^][Cl^−^]

**Figure 5:**
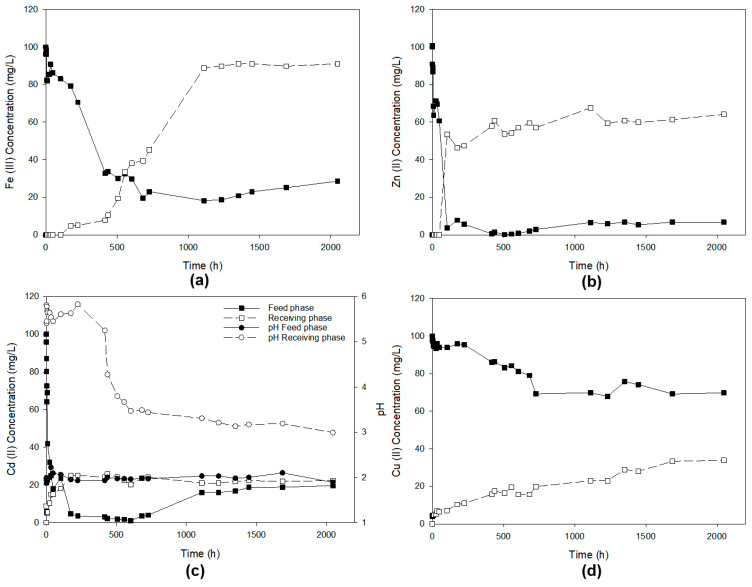
Metal ions concentrations and pH profiles in the feed and receiving phases in the transport of Fe(III) (**a**), Zn(II) (**b**), Cd(II) (**c**) and Cu(II) (**d**) through a PILIM based on [MTOA^+^][Cl^−^]/PVC whit 70% of IL. The feed phase was a mixture of the four metals ions (100 mg/L each) in HCl (1 M). The receiving phase was milli Q water at pH = 6.

As mentioned above, with the ionic liquid [MTOA^+^][Cl^−^], it was possible to prepare membranes with a higher IL/PVC ratio than that used with [omim^+^][PF_6_^−^]. Figure 5 shows the results of the pertraction test of a solution of Fe(III), Zn(II), Cd(II) and Cu(II), with a concentration of 100 ppm of each, dissolved in 1 M HCl, using pH = 6 milli-Q water as the receiving phase.

As it is noticeable in Figure 5a, the extraction of Fe(III) takes place gradually during the first 1100 h of the test and from this point, the concentration of Fe(III) stabilizes in the feed phase, and we could say that high among of Fe(III) is recovered in the receiving phase. The Fe(III) concentration in the receiving phase increases progressively until it stabilizes at 1100 h of operation. The outcome is deemed highly satisfactory despite the substantial duration of the operation. Afterwards, we will deal with the kinetic aspect of the ion-metal separation. Figure 5b shows that practically all the Zn(II) present in the feed solution disappears after about 100 h of operation using a polymeric inclusion membrane based on [MTOA^+^][Cl^−^], but this is not concomitant with the evolution of the Zn(II) concentration in the receiver phase. Figure 5c depicts the concentration profiles of Cd(II) and pH in both the feed and receiving phases under identical operating conditions to that in the previous experiment, using a polymeric inclusion membrane based on [MTOA^+^][Cl^−^].

Interestingly, Cd(II) showed similar behaviour to Zn(II) (Figure 5b), with the difference that the final concentration of Cd(II) in the receiving phase only reached one-third of that reached by Zn(II) during the first 100 h of operation. In the case of Cu permeation (Figure 5d), a continuous decrease in the feed phase concomitant with a continuous increase in the receiving phase is observed. During the experiment, the concentration does not reach the equilibrium between the phase and the receiving phase. As the first observation, we can highlight that much better pertraction results were reached with the membrane based on [MTOA^+^][Cl^−^] than that based on [omim^+^][PF_6_^−^]. This fact could be explained by (i) the higher amount of ionic liquid (active phase) immobilized in the [MTOA^+^][Cl^−^] membrane, (ii) the less amount of PVC (which is inner) in the [MTOA^+^][Cl^−^] membrane and mainly (iii) by the better results in the extraction of the target metal ionic in liquid-liquid experiments as we demonstrated in previous work. In previous work [23], we also studied the selective recovery of Zn(II), Cd(II), Cu(II) and Fe(III) from hydrochloride aqueous solutions (0.1 g/L, HCl-1 M) using [MTOA^+^][Cl^−^]. The extraction capability of [MTOA^+^][Cl^−^] for these metals was near 100% for Cd, Zn and Fe and near 80% for Cu. However, as we commented above, the maximum extraction with [omim^+^][PF_6_^−^] was around 60% in the case of the Cd(II).

**Figure 6:**
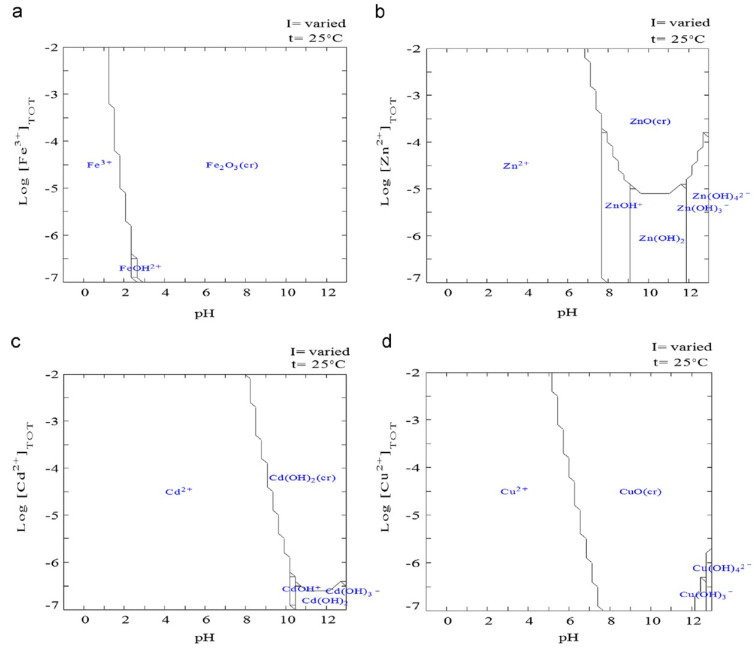
Predominant area diagram for (**a**) Fe(III), (**b**) Zn(II), (**c**) Cd(II) and (**d**) Cu(II) (accessible at https://www.kth.se/che/medusa, accessed on 1 September 2023).

It is worth mentioning that when we do a mass balance between the feed and receiving phases for some of the metal ions, Zn(II) and Cd(II), the total amount of metal in the feed and receiving phase is less than the initial amount of ion metal placed in the fed phase. The decrease in metal ion concentration can be explained by two factors: (i) some of the metal ions have precipitated in either the feed or receiving phase, or (ii) a fraction of the metal ions remains absorbed within the immobilized ionic liquid phase of the membrane. To test these hypotheses, we utilized the software “Medusa v.1” (accessible at https://www.kth.se/che/medusa, accessed on 1 September 2023), which determines the predominant species based on pH and metal ion concentration in the medium. Figure 6 illustrates the diagram of the predominant areas of all the ions present in the hydrochloride solution under study, showing the dominant forms of these metal ions at different concentrations in the medium and pH values (from −1 to 13). Our investigation covered a range of concentrations from log[Mx] = −7 (equivalent to a zero concentration of any metal ion present in either of the two solutions) to log[Mx] = −2, which is the upper limit set since the maximum concentration that can be observed for any metal ion, both in the feed and the receiving solution.

In the case of Zn(II) If we perform a mass balance analysis in the feed and receiving phases, we observe a gradual decrease in Zn(II) concentration over time. Suppose we refer to the Zn(II) predominance diagram (Figure 6b). In that case, we note that Zn(II) initiates precipitation at pH = 7 for high Zn(II) concentrations and pH > 7.7 for low Zn(II) concentrations. These values were not reached during this experimental procedure. Therefore, the detected Zn(II) loss through mass balance cannot be attributed to precipitation. Instead, it could be attributed to the retention of the metal ion on the polymeric inclusion membrane. As Zn(II), the mass balance is not fixed for Cd(II) between the feed and receiving phase. Returning to the specific predominant area diagram for this ion (Figure 6c) and considering the pH profiles obtained, it can be concluded that the Cd(II) ion does not precipitate in the form of hydroxides, but most of it is retained in the polymeric inclusion membrane. These results agree with those observed in previous studies, where the pertraction of the Cd(II) ion has a similar behaviour in supporting liquid membranes based on [MTOA^+^][Cl^−^] [21] to that observed in this study. It is interesting to note that after 1000 h, the concentration of Cd(II) in the feed phase increases, probably due to stripping and redissolution of the ion retained in the membrane. On the contrary, Cd(II) could be stripping in the receiving phase more extensively in PILIMs membranes than in SILMs [19]. 

Regarding the stability of the polymer inclusion membranes, this was weighed before and after the 2048 h (85 days) test. The percentage of ionic liquid remaining in the membrane after the test was 82% of the initial ionic liquid weight. This fact demonstrates the high operational stability of the [MTOA^+^][Cl^−^]/PVC inclusion polymeric membrane at 70% ionic liquid. It is interesting to note that much more [MTOA^+^][Cl^−^] was retained in the pertraction test than after four washing cycles stability test (see before section). In the pertraction experiment, the feed and receiving phases were saturated with the ionic liquid, allowing higher stability in the membrane. 

**Figure 7:**
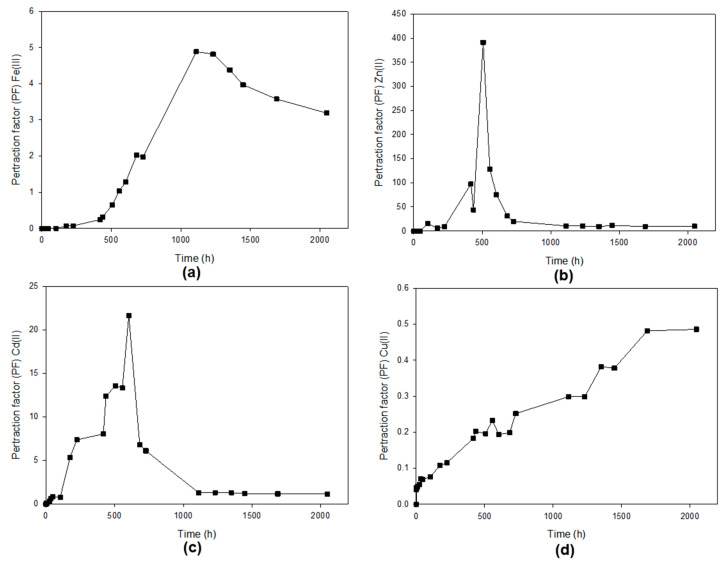
Profile of the pertraction factor (PF) of (**a**) Fe(III), (**b**) Zn(II), (**c**) Cd(II) and (**d**) Cu(II) using a PILIM based on [MTOA^+^][Cl^−^]/PVC whit 70% of IL. The feed phase is a mixture of the four metals ions (100 mg/L each) in HCl (1 M). The receiving phase was milliQ water at pH = 6.

It is also important to point out that the objective of this pertraction process is to achieve the separation of the metal ions in the receiving phase. For this purpose, the pertraction factor (PF) was used to evaluate the recovery of these metal ions in the receiving phase. The pertraction factor could help us to identify the point at which the operation should be stopped to get the maximum separation of the specific ion metal in the striping phase. Figure 7 shows the evolution of the pertraction factor over time for Fe(III), Zn(II), Cd(II) and Cu(II). The higher PF factor was reached by Zn(II) because a high concentration of Zn(II) was reached quickly in the receiving phase.

Meanwhile, the concentration of Zn(II) is drastically reduced in the receiving phase, possibly due to the Zn(II) absorption in the membrane. Cd(II) also reached a high PF due to similar reasons. The concentration of Cd(II) in the feed phase was quickly reduced due to the absorption of Cd(II) in the liquid membrane. Some Cd(II) crosses the membrane towards the receiving phase. The profile observed for Fe(II) is due to the concomitant increase of the concentration in the feed phase with the reduction in the receiving phase. It allows a continuous increase of the PF until 1000 h. In the case of Cu(II), the PF tends to be 1 since the concentrations of Cu(II) tend to be equal on both sides of the membrane.

In the case of Fe(II) (Figure 7a), we can see a gradual increase of this parameter over time, which reaches a maximum value of around 5 at 1100 h. This point represents the optimum operating time for the removal of this ion. In the case of Zn(II) (Figure 7b) the pertraction factor reaches a maximum of 391 at 500 h, indicating the point at which the separation of Zn(II) is highest between the feeding and receiving phases. However, this would not be the optimum point to stop the separation operation. Instead, we must rely on the diagram of the concentrations of the different metal ions (Figure 5) to identify that the correct time to stop the operation would be at 100 h, which would correspond to a pertraction factor of approximately 25, which could be acceptable, but the operation time is reduced considerably.

The maximum Cd(II) pertraction factor, PF = 22, is reached at a time (t = 600 h). Other pertraction factors could be acceptable in less time to reduce the operation time. In the case of Cu(II) ion pertraction, the concentration of Cu(II) in the feed phase decreases continuously while it increases in the receiving phase, although this transport of matter takes place at a low velocity. After more than 2000 h of operation (about 85 days), only 30% of the ion in the feed phase passes to the receiving phase. The concentration of Cu(II) in the receiving phase was never higher than in the feed phase.

As a consequence, as seen in Figure 7, the pertraction factor did not exceed the value of unity. In this case, there is no accumulation of Cu(II) in the membrane since it is observed that the decrease in the concentration of this metal ion in the feeding phase matches the increase of the same in the receiving phase. Furthermore, the diagram of predominant areas for Cu(II) represented in Figure 6d shows that the pH profiles in the experiment do not allow the precipitation of the Cu(II), which makes it possible to satisfy the mass balance for Cu(II) between feed and receiving phase. The results obtained for Cu(II) extraction seem to make sense if we consider that in previous studies carried out under the same conditions as those described for this test, about 80% of Cu(II) was extracted using [MTOA^+^][Cl^−^] in a liquid-liquid extraction [23]. However, when SILMs based on [MTOA^+^][Cl^−^] were used, Cu(II) could not be practically extracted [19]. In liquid-liquid extraction, the ratio of 1 M hydrochloride aqueous solutions to the IL phase was 1 to 1 (*v*/*v*) [23]. In supported ionic liquid membrane experiments, the ration of the feed phase to the ionic liquid phase was approximately 30,000/80 (*v*/*v*) [19]. The amount of occluded ionic liquid was insufficient to extract the metal ions. In the experiment with polymer inclusion membranes based on [MTOA^+^][Cl^−^] (this work), the ration feed phase to ionic liquid phase was approximately 30,000/172 (*v*/*v*), more than the double that in the case of supported liquid membranes which would improve the efficiency of the operation, allowing a partial extraction of the Cu(II) retained in the feed phase, around 30%. It is important to point out that, as commented above, we studied the separation of Fe(III), Zn(II), Cd(II) and Cu(II) through supported ionic liquid membranes based on [MTOA^+^][Cl^−^] in a previous work [19]. The feed phase consisted of a hydrochloride aqueous solution (HCl, 1 M) of the four metal ions at 0.1 g/L. When the receiving phase was milli Q water, the pertraction factor was lower than when polymer inclusion membranes were used with the same phase and receiving phase. The selective separation of Cd(II) and Cu(II) with a polymer inclusion membrane was studied based on CTA and the ionic liquids Cyphos IL 101 and Cyphos IL 104 as carriers. At low HCl concentration, high extraction of Cd(II) was reached, and Cu(II) was almost not extracted in the receiving phase. The increase in the HCl concentration in the feed enhanced Cu(II) extraction and decreased the selectivity coefficient for Cd(II) over Cu(II) [33].

**Figure 8:**
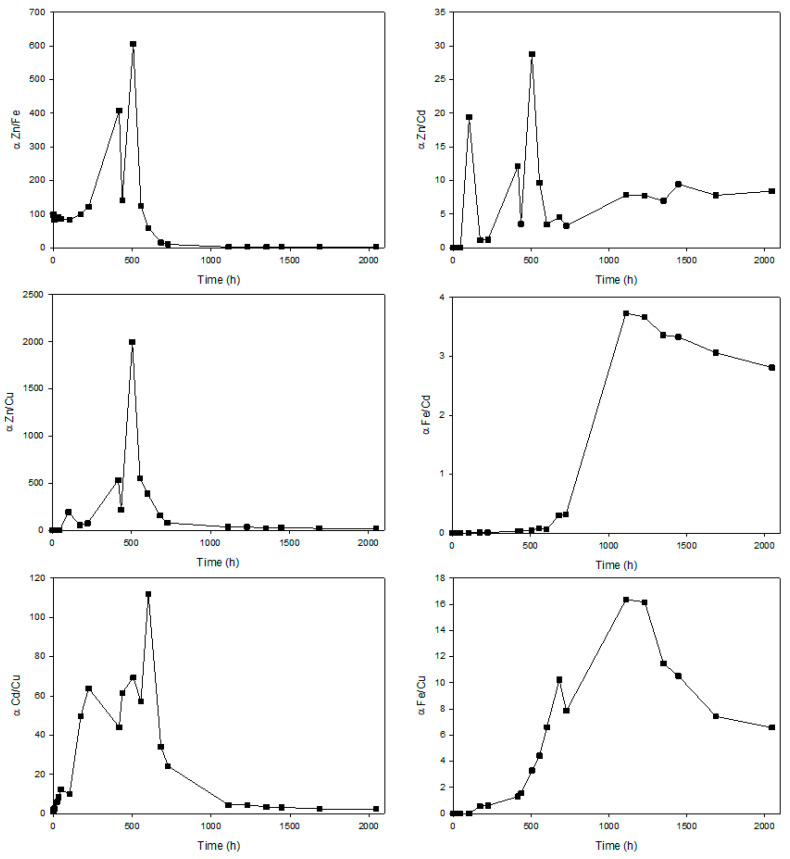
Profile of the separation factors for pairs between Zn(II)/Fe(III), Zn(II)/Cd(II), Zn(II)/Cu(II), Fe(III)/Cd(II), Cd(II)/Cu(II) and Fe(III)/Cu(II) using a PILIM based on [MTOA^+^][Cl^−^]/PVC whit 70% of IL. The feed phase consists of a mixture of the four metals ions (100 mg/L each) in HCl (1 M). The receiving phase was milliQ water at pH = 6.

To gain a deeper understanding of the separation efficiency of a mixture containing Zn(II), Cd(II), Cu(II) and Fe(III) metal ions, we calculated the separation factors (α) for the six possible pairs of these metal ions, which serve as indicators of the efficiency of the selective separation of two metals between the feed and receiving phases. If one metal remains predominantly in the feed phase while the other metal undergoes significant transfer to the receiver phase, then we will be talking about high separation factors. Figure 8 shows the evolution of the separation factors calculated for the six possible pairs of the four metal ions in solution from the concentrations measured in the feed and receiving phase over the test time.

The separation factor reached by the Zn(II)/Cu(II) and Zn(II)/Fe(III) pairs stands out for its high value, with maximums of 1996 and 606, respectively. These high values in the pertraction factor are due to the fact that Zn(II) can diffuse through the PIM based on [MTOA^+^][Cl^−^] and accumulate in the receiving phase. In the case of the Zn(II)/Fe(III) pair, Zn(II) would diffuse faster, allowing an effective separation of Zn(II) at 100 h of operation. At this point, the receiving phase could be renewed and thus recover Fe(III), which has slower diffusion kinetics. Recently, there have been advancements in developing polymer inclusion membranes (PIMs) that incorporate phosphonium-based ionic liquids as carriers. Alongside them, o-nitrophenyloctyl ether acted as a plasticizer, and triacetate cellulose functioned as the polymer matrix. This composite material was formulated and applied for the specific purpose of separating Zn(II) from Fe(III). 1 mol L^−1^ in a hydrochloric acid (HCl) was employed as the stripping phase for Fe(III), facilitating its removal.

Meanwhile, a significant portion of Zn(II) ions were effectively retained in the initial feed phase. The separation factor (SFe(III)/Zn(II)) is 8.85. It should be noted that in this work, they used a continuous operation [34].

In the case of the Zn(II)/Cu(II) pair, the selectivity in the extraction of one compound over the other is even higher than that seen for the Zn(II)/Fe(III) pair. Since the diffusion kinetics of Zn(II) through the membrane are much faster than those of Cu(II), we can point out that there is a possibility of separating Zn(II) from Cu(II) in a successful way. For the Zn(II)/Cd(II) and Fe(III)/Cd(II), relatively high separation factor values 28.8, 3.7 respectively, are obtained, mainly because 80% of the Cd(II) present in the feed phase is retained in the membrane, while the other ions can diffuse to the receiving phase, for the Cd(II)/Cu(II) pair a maximum separation factor of 112 reaches of the low permeability of Cu(II) respect to Cd(II) at short times. For the Fe(III)/Cu(II) pair, high values of the separation factor are reached at the end of the experiment since it is when higher concentrations of Fe(III) are reached in the receiving phase, while Cu(II), which has a slower diffusion rate, remains mainly in the feed phase, due to its slower kinetics to diffuse through the [MTOA^+^][Cl^−^] based polymeric inclusion membrane.

The results obtained in the pertraction test with the [MTOA^+^][Cl^−^]/PVC inclusion membrane were satisfactory in terms of separation capacity. However, the times required for this operation are very high. Considering that after more than 2000 h of testing, the concentrations in the feed and receiving phases were not equalized for any of the ions studied. Furthermore, at the end of the experiment, there was still an HCl gradient of 0.93 M, indicating that equilibrium was not reached. As commented above in a previous work, the selective separation of Fe(III), Zn(II), Cd(II) and Cu(II) through supported ionic liquid membranes was studied. Generally, when supported ionic liquid membranes were used, the separation factor was lower than when polymer inclusion membranes were used. However, the kinetic through polymer inclusion membranes were lower than in the case of supported ionic liquid membranes [19]. The use of polymer inclusion membranes, which are dense, could increase the ions metal selectivity and the membrane stability. However, it reduces the kinetic of the permeation with respect to supported ionic liquid membranes, in which the ionic liquid is just adsorbed on a polymer material. Consequently, the ionic liquid has “more movement ability” than when the IL is occluded in a dense membrane (PILIMs).

The metal ion fluxes through PILMs were calculated from the initial slopes of the metal ion concentration profiles (see Table 1). As observed, the highest fluxes were achieved by Zn(II) and Cd(II) at 10.9 and 9.39 mg m^−2^ h^−1^, respectively. On the other hand, lower fluxes were obtained for Fe(III) and Cu(II) at 1.82 and 2.92 mg m^−2^ h^−1^, respectively.

Recently, the selective separation of Pt(IV), Pd(II), and Rh(III) through polymer ionic liquids inclusion membranes was carried out using different receiving solutions. The PILIM contains the ionic liquids trioctyl(dodecyl) phosphonium chloride (40 wt %), the polymer PVDF-co-HFP (50 wt %), and 2NPOE as plasticizer (10 wt %). The membranes maintained the pertraction factor and the purity of the extract in the receiving phase over the course of 4 cycles (four weeks), demonstrating that the membrane was relatively stable. The flows were 200–1000 mg m^−2^ h^−1^ for more permeable metal, Pt and Pd, respectively, and the transport mechanism was described as ionic exchange [35].

New alkylimidazoliums bromide were tested for the selective separation of Cd(II), Cu(II), Pb(II), and Zn(II) ions from hydrochloride aqueous solutions. The most effective Ionic liquid was the longest alkyl chain length imidazolium ionic liquid and, consequently, the most water-insoluble ionic liquid. The observed permeability order was as follows: Cd(II) > Zn(II) > Pb(II) ≫ Cu(II). Similar to our research work, the permeability of Cd(II) and Zn(II) was higher than the permeability of Cu(II). The increased HCl concentration in the feed solution enhances the Cd ions transport but decreases transport selectivity defined by the relative fluxes [36].

The main limitation of polymer inclusion membranes could be the low kinetic of the ion transport through the membrane. However, in some applications, like membranes in sensors, this kinetic could be sufficient [28]. It would be interesting to consider in future studies different mechanisms to decrease the flow resistance shown by this type of membrane, such as ultrasound or microwave, to improve the permeation rate through the polymeric support.

Regarding the transport mechanism, in the case of [MTOA^+^][Cl^−^], which is a quaternary amine, the extraction mechanism is suggested to involve an ion-exchanged step, as hypothesized by Juang et al. [37] who studied the separation of Zn(II) and Cd(II) from chloride solutions using Aliquat 336, or by Wang et al. [38] who studied the for the separation of the ionic pair Zn(II)/Fe(III) from chloride solutions also using Aliquat 336. The hypothesized mechanism could be represented by the following chemical equation:

[ZnCl_4_^2−^]_(aq)_ + 2[R_3_CH_3_NCl]_(m)_ = 2[R_3_CH_3_N^+^][ZnCl_4_^2−^]_(m)_ + 2Cl^−^ (3)

In contrast, for imidazolium ionic liquids and particularly [omim^+^][PF_6_^−^], it was demonstrated [18] that an increase in HCl concentration enhances the extraction efficiency for Zn(II), Fe(III), and Cd(II). This behavior was not observed in the case of Cu(II). This observation suggests that hydrochloric acid could play a role in extracting metal ions in ionic liquids based on imidazolium cation. In the present experimental context, the immobilization [MTOA^+^][Cl^−^] for extraction Fe(III) at different HCl concentration between the feed phase and receiving phase allow a higher concentration of the metal ion in the receiving phase compared to the equilibrium concentration (50% in each phase). Hence, it is likely that the extraction process will be influenced by the presence of hydrochloric acid in the feed phase. So, the compound formed by the metal ions/IL/hydrochloric acid in the feed phase could be dissociated in the receiving phase due to the lower HCl concentration. Consequently, the transport driving forces are determined by the differences in both metal ion concentration and HCl concentration between the feed and receiving phases. A transport of HCl from the feed to the receiving phase was observed (Figure 4a and Figure 5c), which could be due to the formation of the ion pairs with the IL and the metal ions or the transport of the free HCl by the membrane. Considering the comment above, the ionic nature of ionic liquids can result in various extraction mechanisms, including solvent ion-pair extraction facilitated by HCl, ion exchange, and simultaneous combination of both.

## 4. Conclusions

Polymeric inclusion membranes based on [omim^+^][PF_6_^−^] and [MTOA^+^][Cl^−^] allows more stable membrane than analogous supported ionic liquid membranes towards aqueous phases. Furthermore, it is possible to immobilize more amount of ionic liquids in [omim^+^][PF_6_^−^] and [MTOA^+^][Cl^−^] polymer inclusion membranes than in analogous supported ionic liquid membranes. The optimum ratio of IL/PVC will depend on the nature of the ionic liquid. Despite obtaining very stable polymeric inclusion membranes based on [omim^+^][PF_6_^−^] ionic liquid for IL/PVC ratio 30/70, these membranes failed for the separation of the mixture of the metals under study. On the other hand, with the ionic liquid [MTOA^+^][Cl^−^] and PVC as base polymer, membranes with an IL/PVC ratio of 70/30 were obtained. This membrane allows a high maximum pertraction factor for Zn(II) (391), Cd(II) (22) and Fe(II) (5) and a high separation factor (range from 3 to 2000) for the different ion metal pairs. However, the major drawback of PILIMs is the resistance to matter flow through the membrane.

The high stability of the polymeric inclusion membranes observed in this study and the good performance in separating the metal ions under study make this a promising technology.

## Figures and Tables

**Table 1:** Initial fluxes of metal ions through PILIMs based on [MTOA^+^][Cl^−^]/PVC with 70% of IL.

	Fe(III)	Zn(II)	Cd(II)	Cu(II)
Maximum pertraction factor	4.89	391	21.65	0.49
Initial flux (×10^3^) (g m^−2^ h^−1^)	7.5	21.2	24.9	1.7

## Data Availability

The datasets supporting reported results during the current study are available from the corresponding author upon reasonable request.
